# Supplementary material for: Content-rich biological network constructed by mining PubMed abstracts
Source: BMC Bioinformatics. 2004 Oct 8;5:147. doi: 10.1186/1471-2105-5-147 (PMC528731; doi:10.1186/1471-2105-5-147)
Supplement: Additional File 2 — The original results of the above study (non-essential files are deleted to keep the file size under the limit set by BMC bioinformatics). [file 1471-2105-5-147-S2.bz2 › chilibotAdditionalFile2/dip20/432ID10910894E10362/html/CCR8_CCL17.html]

 


 **CCR8** and **CCL17** 
  
Found 21 abstracts in PubMed, retrieved 20.  
 

 What does Google say? 
 PDF only 
| .edu only 

---

**Interactive relationship** (e.g. stimulation, inhibition, etc)

**Neutral relationship**- These results suggest that TARC  [ **CCL17** ]  utilizes CCR4 and  **CCR8** .  Ref: 10754297 J Immunol, 2000
- ... interactions of CCR4 with MDC CCL22 or TARC  [ **CCL17** ]   **CCL17** ,  **CCR8**  with I 309 CCL1, and CRTH2 with prostaglandin D 2 play a critical role in the allergen induced recruitment of Th2 cells in the target tissues of allergic inflammation.  Ref: 12009564 Mol Immunol, 2002
- Thymus and activation regulated chemokine  [ **CCL17** ]  TARC  [ **CCL17** ]   **CCL17**  is a chemokine that attracts CC chemokine receptor 4 positive CCR4 or  **CCR8**  cells.  Ref: 11240957 J Allergy Clin Immunol, 2001
- TARC  [ **CCL17** ]  interacts primarily with the CCR4 receptor and to a lesser extent with the  **CCR8**  receptor.  Ref: 12832759 Acta Crystallogr D Biol Crystallogr, 2003
- TARC  [ **CCL17** ]  interacts primarily with the CCR4 receptor and to a lesser extent with the  **CCR8**  receptor.  Ref: 12499558 Acta Crystallogr D Biol Crystallogr, 2003
- In vitro polarized human Th2 cells preferentially express the chemokine receptors CCR3, CCR4, and  **CCR8**  and migrate to their ligands eotaxin, monocyte derived chemokine MDC, thymus and activation regulated chemokine  [ **CCL17** ]  TARC  [ **CCL17** ] , and I 309.  Ref: 11390417 J Clin Invest, 2001
- Thymus and activation regulated chemokine  [ **CCL17** ]  TARC  [ **CCL17** ]   **CCL17**  is a member of the CC chemokines and is a chemoattractant for CC chemokine receptor 4 CCR4 and CC chemokine receptor 8  [ **CCR8** ]   **CCR8**  expressing cells.  Ref: 12522366 J Am Acad Dermatol, 2003
- Three human chemokines I 309, thymus and activation regulated chemokine  [ **CCL17** ]  TARC  [ **CCL17** ]  and macrophage inflammatory protein 1beta MIP 1beta have been reported to be ligands for CC chemokine receptor 8  [ **CCR8** ]   **CCR8** .  Ref: 10540332 Eur J Immunol, 1999
- The CC chemokine receptor CCR 8  [ **CCR8** ]  belongs to the seven transmembrane spanning receptor family and functionally responds to the eukaryotic CC chemokines I 309, thymus and activation regulated chemokine  [ **CCL17** ] , macrophage inflammatory protein 1 beta MIP 1b and to the products viral MIP I and viral MIP II of the Kaposi associated herpesvirus HHV 8.  Ref: 10611408 Forum Genova < MedlineTA>Forum Genova , 1999
- Treg cells specifically express the chemokine receptors CCR4 and  **CCR8**  and represent a major subset of circulating CD4 T cells responding to the chemokines macrophage derived chemokine MDC CCL22, thymus and activation regulated chemokine  [ **CCL17** ]  TARC  [ **CCL17** ]   **CCL17** , I 309 CCL1, and to the virokine vMIP I ligands of CCR4 and  **CCR8** .  Ref: 11560999 J Exp Med, 2001
- T regulatory cells, which inhibit dendritic cell function and are probably involved in the termination of ACD, are sensitive to MCP 1, MIPs and TARC  [ **CCL17** ] , but express high levels of  **CCR8**  and are more specifically attracted by I 309.  Ref: 11876523 Arch Dermatol Res, 2002

**Non-interactive relationship** (e.g. studied together, co-existance, homology, etc.)

- In accordance with receptor expression, the response of Th2 cells to I 309  **CCR8**  ligand and thymus and activation regulated chemokine  [ **CCL17** ]  CCR4 and  **CCR8**  ligand is enhanced upon activation.  Ref: 9820476 J Immunol, 1998
- In this report, we present evidence that TARC  [ **CCL17** ]  and MIP 1beta did not bind to or induce chemotaxis through  **CCR8**  on a stable transfected cell line 1D 21 and did not bind to  **CCR8**  on in vitro differentiated human CD4 Th 2 cell cultures.  Ref: 10540332 Eur J Immunol, 1999
- The most active chemokines for resting Th IL 10, in terms of calcium mobilization and in vitro migration, were in order of potency CCL2 monocyte chemoattractant protein 1, CCR2 ligand, CCL4 macrophage inflammatory protein 1beta, CCR5 ligand, CCL3 macrophage inflammatory protein 1alpha, CCR1 5 ligand,  **CCL17**  thymus and activation regulated chemokine  [ **CCL17** ] , CCR4 ligand, CCL1 I 309,  **CCR8**  ligand, CXCL12 stromal derived factor 1, CXCR4, and CCL11 eotaxin, CCR3 ligand.  Ref: 11145678 J Immunol, 2001
- Th2 lymphocytes, which are critical for allergy, employ the CC chemokine receptors CCR4 and  **CCR8**  with the ligands thymus and activation regulated chemokine  [ **CCL17** ]  TARC  [ **CCL17** ] , macrophage derived chemokine MDC and I 309, respectively.  Ref: 11340325 Int Arch Allergy Immunol, 2001
- These data support the role of CCR4, of its ligands MDC and TARC  [ **CCL17** ] , and of  **CCR8**  in the pathogenesis of allergen induced late asthmatic responses and suggest that these molecules could be considered as targets for therapeutic intervention.  Ref: 11390417 J Clin Invest, 2001
- The assignment of chemokine chemokine receptor pairs TARC  [ **CCL17** ]  and MIP 1 beta are not ligands for human CC chemokine receptor 8  [ **CCR8** ] .  Ref: 10540332 Eur J Immunol, 1999
